# Editorialets

**Published:** 1907-03

**Authors:** 


### Editorialets.

THE "RED BACK" is the first to publish the new Medical Practice Act, the one board bill. It passed the House with Senate amendments only finally on the 14th by 92 to 11, and awaits the Governor's signature. Judge Duncan of Smith county, who made a vigorous fight to exempt his old friend, Dr. T., and all osteos who have been practicing five years, and lost out, I understand will lay before the Governor a special plea and protest, "attacking the constitutionality" of the bill.

---

THE PURE FOOD BILL finally passed the House on the 15th with the Senate amendments, the principal one being to locate the Commission at Denton, under the wing of the Girls' Industrial Arts school, instead of in the A. and M. College, as agreed at first by the House. The bill creates a Pure Food Commission consisting of a commissioner and staff of assistants to inspect all food products, liquors and drugs made and sold in Texas. The Federal bill only applies to interstate traffic in such articles. Five thousand dollars is appropriated.

It was thought that this inspection should be done by the Public Health Department, but that department is not in position to handle it, and will not be, unless its power and scope are enlarged and its facilities greatly increased.

As this bill carries an appropriation and is not a "platform demand," its fate is by no means certain. It remains to be seen also what effect fanaticism—sentiment run riot—is going to have on the Governor with regard to the Board of Examiners bill. The Christian Science women will carry the war into the "Africa" of the executive office.

---

DR. R. B. SELLERS has removed from Comanche to Waco. Success to him.

---

FOR SALE.—Good practice and three-room house; five acres of land; two lots and office; in growing town on I. & G. N. R. R. Price, $1500. Address, Dr. J. M. Ware, Magnolia, Texas.

---

THE American Medical Editors' Association will hold its annual meeting at Atlantic City in June, at the time of meeting of the American Medical Association. The membership, under the management of Secretary J. Macdonald, Jr., has grown in a few years from 15 to 140. There is a great work to be done at this meeting to check the encroachments of the Octopus, guided by the apostate homeopath who runs the Association journal—looking to the creation of a great medical journal *trust*, to throttle and kill out the independent medical press. See editorial in this number.

---

A "YEARLY" LIGAMENT.—The *Atlanta Medical Record*, quoting the *American Journal of Surgery*, says: "Never divide the annual ligament of the wrist."

---

THE *American Journal of Clinical Medicine* for March is received. Surely the publishers need not make any excuses for raising the subscription price from $1 to $1.50, for a perusal of the number before us shows quantity, variety and quality not usually found elsewhere, nor at any price. The subscription price at $5 would be reasonable.—*Southern Clinic, Richmond, Va.*

Sure. One of the very best published anywhere.—DANIEL.

MARRIED.—At San Antonio, Texas, March 7 (inst.), Dr. Geo. H. Moody to Miss Bebe Denman, daughter of Judge and Mrs. L. C. Denman, all of San Antonio.

---

DR. JNO S. TURNER, late superintendent of the North Texas State Hospital for Insane, has opened The Arlington Heights Sanitarium at Fort Worth for the treatment of mental and nervous diseases and drug and alcohol addiction. Dr. Turner is an able and efficient neurologist and has had ten years' experience. The State institutions being always crowded, there is demand for a private institution of this kind.

---

*The Journal of Inebriety,* after thirty years of continuous studies of the diseases of inebriety and drug-taking, begins its new decade by entering upon a comparatively new field of physiological and psychological therapeutics, for the treatment of these neuroses. Arrangements have been completed by which *The Archives of Physiological Therapy* has been consolidated and will hereafter be published as a part of *The Journal of Inebriety.* This very able monthly has been developing parallel lines of study with *The Journal of Inebriety.* In the opinion of its managers its scientific value would be greatly enlarged by concentrating its work along some special lines. The disease of inebriety and its allied neuroses is a field of most practical interest, hence *The Journal of Inebriety* is selected as a medium for continuing the work of *The Archives of Physiological Therapy.*

---

A BARBERS' LICENSE LAW.—Senator Paulus has in the Senate a bill to create a board to examine barbers for license. It is framed after the laws now in force, I understand, in Wisconsin, Minnesota, Michigan, Maryland, North Dakota, Utah, Washington, Oregon, Missouri, Kentucky, Maryland, Delaware, New York, Connecticut, New Jersey, and Rhode Island. It is to be hoped the bill will pass. None but doctors appreciate the dangers of an unhygienic barber shop. Skin diseases, especially, spread through the use of razors, brushes and towels that are indiscriminately used.

---

EXAMINATION OF CANDIDATES FOR ASSISTANT SURGEON IN THE PUBLIC HEALTH AND MARINE HOSPITAL SERVICE.—A board of officers will be convened to meet at the Bureau of Public Health and Marine Hospital Service, 3 B street S. E., Washington, D. C., Monday, April 15, 1907, at 10 o'clock a. m., for the purpose of examining candidates for admission to the grade of assistant surgeon in the Public Health and Marine Hospital Service.

Candidates must be between 22 and 30 years of age, graduates of a reputable medical college, and must furnish testimonials from responsible persons as to their professional and moral character.

The following is the usual order of the examinations: 1, physical; 2, oral; 3, written; 4, clinical.

In addition to the physical examination, candidates are required to certify that they believe themselves free from any ailment which would disqualify them for service in any climate.

The examinations are chiefly in writing, and begin with a short autobiography of the candidate. The remainder of the written exercise consists in examination of the various branches of medicine, surgery and hygiene.

The oral examination includes subjects of preliminary education, history, literature and natural sciences.

The clinical examination is conducted at a hospital, and when practicable, candidates are required to perform surgical operations on a cadaver.

Successful candidates will be numbered according to their attainments on examination, and will be commissioned in the same order as vacancies occur.

Upon appointment the young officers are, as a rule, first assigned to duty at one of the large hospitals, as at Boston, New York, New Orleans, Chicago, or San Francisco.

After five years' service, assistant surgeons are entitled to examination for promotion to the grade of passed assistant surgeon.

Promotion to the grade of surgeon is made according to seniority, and after due examination as vacancies occur in that grade.

Assistant surgeons receive $1600, passed assistant surgeons $2000, and surgeons $2500 a year, When quarters are not provided, commutation at the rate of $30, $40, and $50 a month, according to grade, is allowed.

All grades above that of assistant surgeon receive longevity pay, 10 per cent in addition to the regular salary for every five years' service up to 40 per cent after twenty years' service.

The tenure of office is permanent. Officers traveling under orders are allowed actual expenses.

For further information, or for invitation to appear before the board of examiners, address "Surgeon-General, Public Health and Marine Hospital Service, Washington, D. C."

The New Practice Act, while a farce, in that it will permit license to osteopaths to practice all branches of medicine—even obstetrics and those diseases that are known to be amenable to nothing but antiseptic treatment—still is far-reaching for good, and corrects many defects in the old law. It knocks out Christian Science, magnetic healers, vitopaths, "naturo-paths," and the street fakir with his cure-all; and takes account of stock as to all practicers who have qualified under previous acts. By the bye, under what act has any osteopath *ever* qualified? How and when did he ever become a "legal" or "legalized" practitioner? Does *exemption* from the law give one a *legal* standing? This is a nice question for the Attorney General. If *not*, how is one, not a "legal practitioner," eligible to serve on a board, or to practice, without first procuring a license by examination? The law also provides for revoking a license for causes stated.

All in all it is a much better law than we have ever had—and *having* to swallow the osteos—a bitter dose—is a lesser of many evils. I don't think, however, that we have anything to crow about or to be proud of. It is not a credit to regular medicine to mix with the quacks—even though forced upon us.

